# Quality of Life and Psychometric Characteristics of Syrian Refugee Physicians Who Migrated to Turkey: A Cross-Sectional Study

**DOI:** 10.1155/2023/6654937

**Published:** 2023-12-06

**Authors:** Ahmet Keskin, Basri Furkan Dagcioglu

**Affiliations:** Ankara Yildirim Beyazit University, Faculty of Medicine, Department of Family Medicine, Ankara, Türkiye

## Abstract

**Background:**

The concept of migration comes with various problems, affecting the quality of life and psychological state of immigrants. This study aimed to investigate the quality of life and depression and anxiety states of physicians who immigrated to Turkey after the civil war that started in Syria in 2011.

**Methods:**

In this cross-sectional study, a sociodemographic questionnaire form, the short version of the World Health Organization's quality of life assessment tool (WHOQOL-BREF), Beck Depression Inventory (BDI), and Beck Anxiety Inventory (BAI) were applied to Syrian doctors who received integration training to work in refugee health centers established for immigrants in Turkey.

**Results:**

A total of 570 participants were included in the study. The median scores of WHOQOL-BREF domains of the participants were 75 for DOM1 (min: 25, max: 100, IQR: 18), 69 for DOM2 (min: 6, max: 100, IQR: 25), 69 for DOM3 (min: 0, max: 100, IQR: 19), and 63 for DOM4 (min: 0, max: 94, IQR: 19). The median BDI score of the participants was 7 (min: 0, max: 41, IQR: 8), and the median BAI score was 5 (min: 0, max: 50, IQR: 8). Having primary care experience, having knowledge about the Turkish healthcare system, believing that they can adapt to work in refugee health centers, and not having a plan to return to their country were found to be associated with a higher score in at least one of the WHOQOL-BREF subdomains. Planning to turn back their country was significantly associated with higher BAI scores.

**Conclusions:**

The overall quality of life of most refugee physicians in Turkey was high, and the BDI and BAI scores were also below the threshold values. Further qualitative studies that allow in-depth analyses may reveal underlying factors for this situation.

## 1. Introduction

The civil war that began in Syria in 2011 has led to the forced migration of the Syrian population to other countries [1]. Turkey, one of the first and most affected countries by this migration, has had to host more than 3.5 million Syrian refugees [2]. In order to find solutions to the health needs of these immigrants, the Turkish government has granted them broad rights in health, education, and employment by placing them under “temporary protection” status [3]. The government also established refugee health centers and enabled Syrian refugee doctors to provide services to their citizens [4]. This project aimed to overcome the language barrier and social security problem, which are significant barriers for immigrants in accessing health care, and to provide employment opportunities for Syrian refugee doctors, who can be described as highly qualified refugees. The primary aim of these centers was to provide primary healthcare services. Initially, it was planned that all Syrian doctors would work as primary care doctors, regardless of their specialty, and extensive adaptation training was organized for this purpose [4].

Many studies in the literature have shown that the concept of immigration causes various problems in many aspects [5]. It has been linked to quality of life and psychological problems such as posttraumatic stress disorder and anxiety [6]. Moreover, such problems have been found to exist even among highly educated immigrants and even in the case of immigration to developed countries [7–9]. This study aims to investigate the quality of life and some psychometric characteristics of Syrian physicians who migrated to Turkey as highly qualified refugees and examine the factors associated with these parameters.

## 2. Methods

### 2.1. Study Design

A cross-sectional study design was chosen.

### 2.2. Participants and Eligibility Criteria

Between November 2016 and April 2018, a sum of 1,095 physicians who participated in “Syrian Physicians' Adaptation Training” were considered potentially eligible for the study. Among these, 570 physicians confirmed eligible, who accepted to participate in the study by reading the informed consent form and could read and write in English adequately enough to maintain the minimum standards to fill the forms, underwent a structured questionnaire. The survey form included sociodemographic data, the World Health Organization Quality of Life (WHOQOL-BREF) questionnaire, the Beck Depression Inventory (BDI), and the Beck Anxiety Inventory (BAI). All survey forms were in English. The power analysis revealed that the sample included in the study reflected the entire population with a 95% confidence level and less than a 3% margin of error.

### 2.3. Data Collection Instruments

#### 2.3.1. Sociodemographic Questionnaire

In the first section, 18 sociodemographic questions were asked to the participants, including age, sex, marital status, having children, duration of living in Turkey, persons living with, place of residence, specialty in medicine, duration of working as a doctor, previous workplace, occupational status, experience in primary care, having information about Turkish Health System, expectations from the Turkish government, opinions on adapting to work in Refugee Health Centers, and plans about turning back to their country.

#### 2.3.2. WHOQOL-BREF

WHOQOL-BREF tool assesses individuals' perceptions of how their life positions coincide with their goals, expectations, and concerns. It is the abbreviated form of WHOQoL-100, developed by the World Health Organization's WHOQoL study group in 15 different cultural settings through years of collective work, encompassing a hundred questions about 24 facets of overall quality of life and general health [10]. In this context, WHOQOL-BREF consists of 26 questions and offers the opportunity to evaluate four main domains: physical health, psychological, social relationships, and environment (Table 1). Validity studies showed that domain scores produced by the WHOQOL-BREF correlate highly (0.89 or above) with WHOQoL-100 domain scores [10].

The specific calculation method is used for scoring WHOQOL-BREF as defined in the test instructions, and the domains' scores were transformed to a 0–100 scale [11]. The higher scores showed a higher quality of life; a score of 60 was accepted as the cutoff value for a high quality of life [12].

#### 2.3.3. Beck Depression Inventory (BDI)

The BDI is a well-known 21-item self-report scale used to assess the severity of depression in normal and psychiatric populations. It was first developed by Beck et al. in 1961 and revised in 1996 [13]. The scores that can be obtained from the inventory range from 0 to 63, and in the interpretation of the test score, 0–9 points show minimal depression, 10–16 points show mild depression, 17–29 points show moderate depression, and 30–63 points show severe depression [14]. In our study, the score of 17 was accepted as the cutoff value for depression in the binary grouping as normal or depressive.

#### 2.3.4. Beck Anxiety Inventory (BAI)

The BAI is also a 21-item handy self-report scale widely used to assess the severity of anxiety. The scores that can be obtained from the inventory range from 0 to 63, and in the interpretation of the test scores, 0–7 points mean no significant anxiety, 8–15 points show minimal anxiety, 16–25 points show moderate anxiety, and 26–63 points show severe anxiety [15]. In our study, the score of 16 was accepted as the cutoff value for anxiety in the binary grouping as normal or anxious.

### 2.4. Statistical Analysis

Numbers and percentages were used to represent descriptive data. The Kolmogorov–Smirnov test was used to examine the normal distribution of data. The mean ± standard deviation was given for the normally distributed data, and the median and minimum-maximum values besides interquartile range (IQR) were given for the data that did not distribute normally. The chi-square test and adjusted residual analysis were preferred in comparing categorical variables and the binary WHOQOL-BREF grouping. The Spearman correlation test was used to compare continuous numerical data between groups. The Kruskal–Wallis test was preferred to analyze independent variables. The IBM SPSS v.20 package program was used for all statistical analyses, and *p* < 0.05 was accepted as the limit of alpha error.

## 3. Results

The median age of the 570 participants included in the study was 40 years (min: 22, max: 84, IQR: 16). Of the participants, 496 (87%) were married, 480 (84.2%) had children, and the median number of children was 3 (min: 1, max: 9, IQR: 2). Thirteen (2.7%) participants were living alone, although they had children.

The median scores of WHOQOL-BREF domains of the participants were 75 (min: 25, max: 100, IQR: 18) for DOM1, 69 (min: 6, max: 100, IQR: 25) for DOM2, 69 (min: 0, max: 100, IQR: 19) for DOM3, and 63 (min: 0, max: 94, IQR: 19) for DOM4. The association of WHOQOL-BREF transformed domain scores with sociodemographic variables is shown in Table 2. In the binary grouping based on the accepted cutoff value of 60, of all participants, 83.6% for DOM1, 69.9% for DOM2, 64.5% for DOM3, and 54.9% for DOM4 were found to have a normal or high quality of life. The association of binary-grouped WHOQOL-BREF domain scores with sociodemographic variables is shown in Table 3.

The median BDI score of the participants was 7 (min: 0, max: 41, IQR: 8), and the median BAI score was 5 (min: 0, max: 50, IQR: 8). While 10.4% of all participants had moderate or severe depression scores, 14.9% of them were classified as moderate or severe anxiety. The association of total BDI and BAI scores with sociodemographic variables is given in Table 4. The association of binary-grouped BDI and BAI scores with sociodemographic variables is given in Table 5.

A moderate inverse correlation was found between the BDI score and WHOQOL-BREF domain scores. A similar inverse correlation was prominent between the BAI score and DOM1 and DOM2 scores. The correlation analysis results between BDI, BAI, and WHOQOL-BREF domain scores are given in Table 6.

## 4. Discussion

This study on a special group of refugee doctors, the like of which is rare in the literature, reveals some interesting results. The doctors in the study had been forced to leave their country because of civil war. Therefore, low quality of life and high levels of depression and anxiety might be expected in such a vulnerable group. Interestingly, our study showed that the mean quality-of-life scores were mostly above the threshold. Similarly, no significant depression or anxiety was found in the majority of participants.

The negative impact of migration on mental health is well documented. A systematic review of the mental health status of Syrian migrants examined 64 studies, focusing mainly on the prevalence of posttraumatic stress disorder (PTSD), depression, and anxiety, with varying rates. Other outcomes examined included challenges in the postmigration period and factors that promote mental health, such as resilience, positive coping strategies, and psychosocial well-being. As a result, studies have shown a high prevalence of mental health disorders among refugees [16]. However, a study of Syrian refugee doctors found that the level of social adaptation of Syrian doctors living in Turkey was high and highlighted the cultural similarities between the two countries and the extensive social rights granted to Syrian refugees by the Turkish government as possible reasons for this situation [17]. Besides social adaptation, these reasons and the historical and geographical proximity of the two countries may have positively impacted the quality of life of the refugee doctors and may also be effective in reducing depression and anxiety levels. The literature also reports that the quality-of-life scores of health professionals working in migrant health centers in Şanlıurfa, one of the cities with a large population of Syrian refugees in Turkey, were also higher than expected [18]. Another substantial study argued that Turkey's immigration policy is quite comprehensive and effective compared to the policies adopted by various countries to integrate immigrants. The study highlighted the importance of Turkey's initiatives in subjects such as recognition of legal residence, employment, housing, education, public assistance, security, health care, family unity, and others for the integration of migrants [19]. Given these findings, it can be concluded from the relevant literature that the quality of life of Syrian refugee doctors in Turkey is relatively high compared to those in similar situations [20–22].

Regarding the parameters related to quality of life, we found that having experience in primary care, having knowledge about the Turkish health system, believing that they can adapt to working in refugee health centers, and not having a plan to return to their country were associated with higher scores in at least one of the WHOQOL-BREF subdomains. The fact that the health system in Turkey has been transformed to meet the needs of Syrian migrants is likely to be one of the reasons for these findings [23]. In this context, priority has been given to primary health care, which is one of the leading health needs of migrants, and Syrian refugee doctors have been allowed to work mainly in refugee health centers [23, 24]. A recent study of refugee doctors in Turkey reported that refugee doctors, defined as qualified refugees, face difficulties in obtaining equivalence of medical qualifications and that the employment of specialists from different fields as general practitioners can lead to deskilling or overqualification [3]. It was also noted that the participants had different resistance capacities to cope with this situation [3]. This may explain the different responses to the phenomenon of forced migration. Other studies have reported similar problems for highly skilled refugees, even in high-income countries such as Norway and the USA [25, 26].

In addition, married participants and those living in an extended family had higher scores in the social subdomain (DOM3). This result is not surprising, given the association of these parameters with social life. Thus, studies show a significant relationship between marital status, family status, and social quality of life. For example, a study of Syrian refugees in Sweden highlights the significant impact of social support on overall health [27]. Another study conducted in Norway suggests the positive impact of the social environment on the health-related quality of life of young Syrian refugees [28]. Ermanson et al. conducted a comprehensive review of the impact of postmigration factors on the mental health of refugees. It included 34 studies in high-income countries and highlighted the role of place characteristics in facilities, neighborhoods, and countries. Despite limited theorization, all studies indicate a strong association between place of residence and refugee mental health outcomes [29]. Another systematic review on this topic focused on the health-related quality of life of refugees living in the host community and found that factors such as lower employment rates, income, loss of social networks, limited access to health care, and higher rates of mental disorders contribute to their lower quality of life. Comparing the two groups, the general refugee population has higher scores in the physical domain but lower scores in the environmental domain. In comparison, the clinical refugee group (selected specifically because of their mental status or because they had experienced relevant trauma in the past) has higher scores in the environmental domain but lower scores in the psychological domain. These results highlight the complexity of the refugee experience and the need for comprehensive support [30].

When we analyzed the relationship between depression and anxiety with the variables we examined, we saw that female participants and those who were apprehensive about adjusting to working in refugee health centers had higher anxiety scores. Those who did not have primary care experience and those who planned to return to their country had higher depression and anxiety scores. However, in the binary grouping analysis based on the BDI and BAI cutoff scores, the most significant relationship was observed between those planning to return to their country and those with high anxiety scores. This finding primarily suggests that uncertainty about the future may be related to depression and anxiety, although causality cannot be inferred in our study design. Nevertheless, it is noteworthy that most participants did not have scores indicating significant depression and anxiety. In this context, Topaloğlu's study of Syrian refugee doctors in Turkey is fascinating in that it documents that despite all the different characteristics of the participants, such as experience, specialty, age, gender, and official status, they perceive themselves as Syrian doctors rather than Syrian refugees [3]. This situation leads us to believe that the Syrian refugee doctors' physician identity overrides their refugee identity. They do not feel like foreigners in Turkey as respected physicians serving their refugee citizens, which may also be associated with lower-than-expected BDI and BAI scores.

The correlation analysis in Table 6 shows that there was a significant relationship between the BDI, BAI, and all WHOQOL-BREF domains, with the most significant association being between DOM1, which indicates physical quality of life, and DOM2, which indicates psychological quality of life. Studies of similar populations in the literature show a strong relationship between psychiatric status and QOL [31–33]. While this finding reiterates the importance of mental and physical integrity, it also reminds us that a bio-psychosocial approach is essential, especially for vulnerable groups such as refugees [29, 34].

### 4.1. Limitations

Since the questionnaires and inventories used were in English, which was not the native language of the participants, only participants with a sufficient level of English were included in the study. It was found that most of the participants had sufficient knowledge of English to answer the questionnaire, and that lack of fluency in English was a reason for refusing to participate in the study for only a few of them. However, the reasons for not participating in the study were not investigated in detail. This situation could be a possible confounding factor affecting the external validity of the study.

## 5. Conclusions

The overall quality of life of most refugee physicians in Turkey was high, and the BDI and BAI scores were also below the threshold values. Among the possible explanations for this situation, we believe that the transformation of the healthcare system in Turkey to meet the needs of immigrants, the opportunity for refugee doctors to provide health care to their own citizens, and the historical and cultural similarities between the two countries could be considered. The determinants of migrants' quality of life are complex and depend on the integration policies of governments, the contribution of nongovernmental organizations, and other factors [19, 35]. However, qualitative studies that allow in-depth analyses are needed to clarify this situation.

## Figures and Tables

**Table 1:** The main domains and associated facets of WHOQOL-BREF [10].

Domains	Associated facets
Domain 1 (physical health)	Pain and discomfort
	Sleep and rest
	Energy and fatigue
	Mobility
	Activities of daily living
	Dependence on medicinal substances and medical aids
	Work capacity

Domain 2 (psychological)	Positive feelings
	Thinking, learning, memory, and concentration
	Self-esteem
	Bodily image and appearance
	Negative feelings
	Spirituality/religion/personal beliefs

Domain 3 (social relationships)	Personal relationships
	Social support
	Sexual activity

Domain 4 (environment)	Freedom, physical safety, and security
	Home environment
	Financial resources
	Health and social care: accessibility and quality
	Opportunities for acquiring new information and skills
	Participation in and opportunities for recreation/leisure activity
	Physical environment (pollution/noise/traffic/climate)
	Transport

**Table 2:** The association of WHOQOL-BREF transformed scores with sociodemographic variables.

Variables	WHOQOL-BREF score (transformed to 0–100 scale)											
	DOM1			DOM2			DOM3			DOM4		
	Mean/median	Min-max(IQR)	*p*	Mean/median	Min-max(IQR)	*p*	Mean/median	Min-max(IQR)	*p*	Mean/median	Min-max(IQR)	*p*
*Gender*												
Female	71.7/69	25–100 (18)	0.064	66.9/69	6–100 (25)	0.638	65.1/69	6–100 (25)	0.104	62.5/63	6–94 (25)	0.096
Male	73.9/75	31–100 (12)		68.1/69	19–100 (25)		67.3/69	0–100 (19)		59.4/63	0–94 (19)	

*Marital status*												
Married	73.7/75	25–100 (18)	0.625	68.2/69	6–100 (25)	0.121	67.5/69	0–100 (19)	0.058	59.8/63	0–94 (19)	0.429
Other (single, widowed, divorced)	72.1/75	38–100 (25)		64.9/69	31–100 (19)		61.7/63	19–100 (25)		61.5/63	38–94 (19)	

*Family status*												
Core family	73.4/75	25–100 (18)	0.198	67.8/69	13–100 (25)	0.258	67/69	0–100 (19)	**0.029**	59.8/63	0–94 (19)	0.657
Extended family	77/81	38–100 (199)		70.7/69	6–100 (18)		72/75	6–100 (12)		60.6/69	6–94 (25)	
Alone/other	71.4/75	38–94 (12)		65.6/69	31–94 (19)		61.5/56	19–100 (25)		62/63	38–94 (19)	

*Having children*												
Yes	73.5/75	31–100 (18)	0.977	68/69	19–100 (25)	0.771	67/69	0–100 (19)	0.461	59.5/63	0–94 (19)	0.069
No	73.3/75	25–100 (21)		67/69	6–100 (25)		65.6/69	6–94 (21)		62.8/63	6–94 (19)	

*Place of residence*												
City center	73.3/75	31–100 (18)	0.163	67.7/69	6–100 (25)	0.060	66.7/69	0–100 (19)	0.144	59.9/63	0–94 (19)	0.382
County	73/75	25–100 (15)		66.7/69	13–100 (25)		65.9/69	25–100 (28)		59.6/63	19–94 (19)	
Village	80.6/81	63–100 (19)		76.7/75	56–94 (19)		76.1/75	50–100 (19)		64.8/63	50–81 (13)	

*Status of the house of living*												
Renter	73.6/75	25–100 (14)	0.742	67.9/69	6–100 (25)	0.691	67/69	0–100 (19)	0.812	60/63	0–94 (19)	0.332
Owner	74.7/75	44–94 (19)		69.3/69	31–94 (25)		67.7/69	25–100 (31)		62.7/63	31–81 (19)	
Other (living in the house of a relative/friend, etc.)	69.3/69	38–94 (30)		63.9/63	31–94 (39)		63.4/69	19–100 (25)		57.6/63	31–75 (22)	

*Specialty*												
General practitioner	73.3/75	25–100 (18)	0.906	67.1/69	6–100 (25)	0.826	66.7/69	0–100 (25)	0.946	59.6/63	0–94 (19)	0.628
Specialist	73.5/75	31–100 (18)		68.1/69	19–100 (25)		66.9/69	0–100 (19)		60.2/63	13–94 (19)	

*Primary care experience*												
Yes	74.4/75	25–100 (12)	**0.012**	68.7/69	6–100 (25)	**0.002**	67.1/69	0–100 (19)	0.224	60.1/63	0–94 (19)	0.956
No	69.5/69	38–100 (18)		64/63	25–100 (19)		65.7/69	19–94 (25)		59.7/63	19–88 (18)	

*Having knowledge about Turkish healthcare system*												
Yes	75.1/75	31–100 (19)	0.195	70/69	6–100 (18)	**0.009**	68.2/75	6–100 (25)	0.076	61.2/63	6–94 (19)	0.474
No	73/75	25–100 (18)		67.1/69	13–100 (25)		66.4/69	0–100 (19)		59.6/63	0–94 (19)	

*Can adapt to work in refugee health centers?*												
Yes	74.5/75	31–100 (12)	**0.006**	69.2/69	6–100 (25)	**<0.001**	67.8/69	0–100 (22)	**0.016**	60.5/63	0–94 (19)	0.422
No/doubtful	70.6/69	25–100 (18)		63.8/69	13–100 (19)		64.2/69	0–100 (25)		58.7/63	13–88 (19)	

*Planning to go back to his/her own country?*												
Yes or perhaps	71.9/75	25–100 (18)	**<0.001**	66.3/69	13–100 (19)	**0.001**	65.7/69	0–100 (25)	0.060	58.7/63	0–94 (19)	**0.005**
No	76.1/81	31–100 (19)		70.4/75	6–100 (18)		69/75	0–100 (25)		63/62.2	6–94 (13)	

DOM1: domain 1 (physical health), DOM2: domain 2 (psychological), DOM3: domain 3 (social relationships), DOM4: domain 4 (environment), min: minimum, max: maximum, and IQR: interquartile range. The values significant at *p* < 0.05 level are shown in bold.

**Table 3:** The association of binary grouped WHOQOL-BREF domain scores with sociodemographic variables.

Variables		*n* (%)^a^	Binary WHOQOL-BREF domain score grouping											
			DOM1 (*n* = 566)			DOM2 (*n* = 565)			DOM3 (*n* = 569)			DOM4 (*n* = 568)		
			Low *n* (%)^b^	Normal or high *n* (%)^b^	*p*(*X*^2^)	Low *n* (%)^b^	Normal or high *n* (%)^b^	*p*(*X*^2^)	Low *n* (%)^b^	Normal or high *n* (%)^b^	*p*(*X*^2^)	Low *n* (%)^b^	Normal or high *n* (%)^b^	*p*(*X*^2^)
*Gender*														
Female	119 (20.9)		24 (20.3)	94 (79.7)	0.210 (1.658)	36 (30.5)	82 (69.5)	0.911 (0.013)	45 (38.1)	73 (61.9)	0.518 (0.451)	45 (37.8)	74 (62.2)	0.079 (3.201)
Male	451 (79.1)		69 (15.4)	379 (84.6)		134 (30)	313 (70)		157 (34.8)	294 (65.2)		211 (47)	238 (53)	

*Marital status*														
Married	496 (87)		79 (16.1)	413 (83.9)	0.505 (0.384)	141 (28.7)	350 (71.3)	0.077 (3.353)	167 (33.7)	328 (66.3)	**0.027** (5.169)	226 (45.7)	268 (54.3)	0.453 (0.705)
Other (single, widowed, divorced)	74 (13)		14 (18.9)	60 (81.1)		29 (39.2)	45 (60.8)		35 (47.3)	39 (52.7)		30 (40.5)	44 (59.5)	

*Family status*														
Core family	476 (84.1)		80 (16.9)	392 (83.1)	0.501 (1.384)	143 (30.3)	329 (69.7)	0.258 (2.707)	166 (34.9)	309 (65.1)	**0.015** (8.463)	218 (46)	256 (54)	0.293 (2.452)
Extended family	38 (6.7)		4 (10.5)	34 (89.5)		8 (21.1)	30 (78.9)		9 (23.7)	29 (76.3)		17 (44.7)	21 (55.3)	
Alone/other	52 (9.2)		7 (13.5)	45 (86.5)		19 (37.3)	32 (62.7)		27 (51.9)	25 (48.1)		18 (34.6)	34 (65.4)	

*Having children*														
Yes	480 (84.2)		77 (16.2)	399 (83.8)	0.756 (0.141)	138 (29.1)	337 (70.9)	0.259 (1.521)	166 (34.7)	313 (65.3)	0.339 (0.945)	222 (46.4)	256 (53.6)	0.135 (2.297)
No	90 (15.8)		16 (17.8)	74 (82.2)		32 (35.6)	58 (64.4)		36 (40)	54 (60)		34 (37.8)	56 (62.2)	

*Place of residence*														
City center	447 (80.5)		73 (16.5)	370 (83.5)	0.389 (1.887)	135 (30.5)	307 (69.5)	0.046 (6.151)	156 (35)	290 (65)	0.152 (3.773)	202 (45.4)	243 (54.6)	0.456 (1.572)
County	89 (16)		16 (18)	73 (82)		30 (33.7)	59 (66.3)		38 (42.7)	51 (57.3)		42 (47.2)	47 (52.8)	
Village	19 (3.4)		1 (5.3)	18 (94.7)		1 (5.3)	18 (94.7)		4 (21.1)	15 (78.9)		6 (31.6)	13 (68.4)	

*Status of the house of living*														
Renter	504 (92.1)		80 (16)	420 (84)	0.202 (3.198)	148 (29.5)	353 (70.5)	0.776 (0.507)	178 (35.4)	325 (64.6)	0.789 (0.474)	229 (45.6)	273 (54.4)	0.265 (2.656)
Owner	27 (4.9)		3 (11.1)	24 (88.9)		7 (28)	18 (72)		8 (29.6)	19 (70.4)		8 (29.6)	19 (70.4)	
Other (living in the house of a relative/friend, etc.)	16 (2.9)		5 (31.3)	11 (68.8)		6 (37.5)	10 (62.5)		5 (31.3)	11 (68.8)		7 (43.8)	9 (56.3)	

*Specialty*														
General practitioner	164 (29.2)		27 (16.7)	135 (83.3)	0.900 (0.018)	50 (30.7)	113 (69.3)	0.839 (0.832)	60 (36.6)	104 (63.4)	0.698 (0.201)	76 (46.3)	88 (53.7)	0.710 (0.149)
Specialist	397 (70.8)		64 (16.2)	331 (83.8)		117 (29.8)	276 (70.2)		137 (34.6)	259 (65.4)		176 (44.6)	219 (55.4)	

*Primary care experience*														
Yes	452 (81.6)		68 (15.2)	380 (84.8)	0.077 (3.269)	126 (28.2)	321 (71.8)	0.073 (3.259)	158 (35)	293 (65)	0.818 (0.106)	200 (44.4)	250 (55.6)	0.441 (0.702)
No	102 (18.4)		23 (22.5)	79 (77.5)		38 (37.3)	64 (62.7)		34 (33.3)	68 (66.7)		50 (49)	52 (51)	

*Having knowledge about Turkish healthcare system*														
Yes	138 (24.6)		17 (12.5)	119 (87.5)	0.184 (2.077)	29 (21)	109 (79)	**0.008** (7.027)	43 (31.4)	94 (68.6)	0.260 (1.322)	58 (42)	80 (58)	0.431 (0.733)
No	424 (74.4)		75 (17.8)	347 (82.2)		138 (32.99	281 (67.1)		156 (36.8)	268 (63.2)		195 (46.2)	227 (53.8)	

*Can adapt to work in refugee health centers?*														
Yes	415 (74.1)		59 (14.3)	354 (85.7)	**0.048** (4.279)	114 (27.8)	296 (72.2)	0.073 (3.317)	140 (33.8)	274 (66.2)	0.191 (1.795)	182 (44)	232 (56)	0.211 (0.807)
No/doubtful	145 (25.9)		31 (21.7)	112 (78.3)		52 (35.9)	93 (64.1)		58 (40)	87 (60)		70 (48.3)	75 (51.7)	

*Planning to go back to his/her own country?*														
Yes or perhaps	351 (62.5)		63 (18.1)	286 (81.9)	0.092 (2.075)	116 (33.2)	233 (66.8)	**0.035** (4.719)	133 (37.9)	218 (62.1)	0.145 (2.398)	171 (48.9)	179 (51.1)	**0.018** (5.831)
No	211 (37.5)		28 (13.4)	181 (86.6)		51 (24.5)	157 (75.5)		66 (31.4)	144 (68.6)		81 (38.4)	130 (61.6)	

*X* ^2^: chi-square, DOM1: domain 1 (physical health), DOM2: domain 2 (psychological), DOM3: domain 3 (social relationships), and DOM4: domain 4 (environment). ^a^Column percentage. ^b^Row percentage. The values significant at *p* < 0.05 level are shown in bold.

**Table 4:** The association of total BDI and BAI scores with sociodemographic variables.

Variables	BDI score			BAI score		
	Median	Min-max (IQR)	*P* (*Z*or *X*^2^)	Median	Min-max (IQR)	*P* (*Z*)
*Gender*						
Female	7	0–41 (9)	0.526 (−0.635)	7	0–50 (11)	**0.014** (−2.452)
Male	7	0–40 (8)		5	0–50 (7)	

*Marital status*						
Married	7	0–41 (8)	0.935 (−0.081)	5	0–50 (8)	0.395 (−0.851)
Other (single, widowed, divorced)	7.5	0–35 (9)		6	0–48 (11)	

*Family status*						
Core family	7	0–41 (8)	0.575 (1.108)	5	0–50 (8)	0.098 (4.637)
Extended family	6	0–32 (10)		3	0–27 (9)	
Alone/other	8	0–35 (10)		6	0–48 (9)	

*Having children*						
Yes	7	0–41 (8)	0.720 (−0.358)	5	0–50 (8)	0.922 (−0.098)
No	8	0–35 (8)		5	0–48 (9)	

*Place of residence*						
City center	7	0–41 (8)	0.467 (1.524)	5.5	0–50 (9)	0.743 (0.593)
County	7	0–37 (8)		5	0–41 (9)	
Village	5	0–23 (6)		3	0–28 (10)	

*Status of the house of living*						
Renter	7	0–40 (8)	0.100 (4.599)	5	0–50 (8)	0.644 (0.880)
Owner	6	0–21 (8)		7	0–25 (7)	
Other (living in the house of a relative/friend, etc.)	11	1–41 (12)		5	0–50 (5)	

*Specialty*						
General practitioner	6.5	0–40 (8)	0.890 (−0.138)	5.5	0–46 (10)	0.894 (−0.134)
Specialist	7	0–41 (9)		5	0–50 (8)	

*Primary care experience*						
Yes	6	0–41 (8)	**0.003** (−3.006)	5	0–50 (8)	**0.037** (−2.084)
No	9	0–40 (8)		6	0–50 (8)	

*Having knowledge about Turkish healthcare system*						
Yes	6	0–41 (8)	0.087 (−1.712)	5	0–43 (7)	0.171 (−1.369)
No	7	0–40 (9)		6	0–50 (9)	

*Can adapt to work in refugee health centers?*						
Yes	7	0–40 (8)	0.182 (−1.335)	5	0–50 (9)	**0.010** (−2.586)
No/doubtful	7	0–41 (10)		6	0–50 (8)	

*Planning to go back to his/her own country?*						
Yes or perhaps	7	0–41 (10)	**0.002** (−3.075)	6	0–50 (9)	**0.003** (−2.979)
No	6	0–35 (7)		5	0–50 (8)	

BDI: Beck Depression Inventory, BAI: Beck Anxiety Inventory, min: minimum, max: maximum, and *X*^2^: chi-square. The values significant at *p* < 0.05 level are shown in bold.

**Table 5:** The association of binary grouped BDI and BAI scores with sociodemographic variables.

Variables	*n* (%)	BDI score grouping			BAI score grouping		
		Normal/mild depression (*n* = 498)	Moderate/severe depression (*n* = 58)	*p* (*X*^2^)	Normal/mild anxiety (*n* = 480)	Moderate/severe anxiety (*n* = 84)	*p* (*X*^2^)
*Gender*							
Female	119 (20.9)	100 (86.2)	16 (13.8)	0.231 (1.773)	96 (80.7)	23 (19.3)	0.147 (2.339)
Male	451 (79.1)	398 (90.5)	42 (9.5)		384 (86.3)	61 (13.7)	

*Marital status*							
Married	496 (87)	435 (90.2)	47 (9.8)	0.130 (1.796)	420 (85.7)	70 (14.3)	0.190 (1.089)
Other (single, widowed, divorced)	74 (13)	63 (85.1)	11 (14.9)		60 (81.1)	14 (18.9)	

*Family status*							
Core family	476 (84.1)	420 (90.5)	44 (9.5)	0.162 (3.638)	401 (85.3)	69 (14.7)	0.506 (1.363)
Extended family	38 (6.7)	32 (86.5)	5 (13.5)		34 (89.5)	4 (10.5)	
Alone/other	52 (9.2)	42 (82.4)	9 (17.6)		42 (80.8)	10 (19.2)	

*Having children*							
Yes	480 (84.2)	419 (89.99)	47 (10.1)	0.572 (0.368)	407 (85.7)	68 (14.3)	0.417 (0.793)
No	90 (15.8)	79 (87.8)	11 (12.2)		73 (82)	16 (18)	

*Place of residence*							
City center	447 (80.5)	391 (89.1)	48 (10.9)	0.679 (0.775)	375 (84.7)	68 (15.3)	0.844 (0.339)
County	89 (16)	78 (90.7)	8 (9.3)		75 (85.2)	13 (14.8)	
Village	19 (3.4)	18 (94.7)	1 (5.3)		17 (89.5)	2 (10.5)	

*Status of the house of living*							
Renter	504 (92.1)	441 (89.6)	51 (10.4)	0.063 (5.514)	421 (84.5)	77 (15.5)	0.237 (2.879)
Owner	27 (4.9)	26 (96.3)	1 (3.7)		26 (96.3)	1 (3.7)	
Other (living in the house of a relative/friend, etc.)	16 (2.9)	11 (73.3)	4 (26.7)		14 (87.5)	2 (12.5)	

*Specialty*							
General practitioner	164 (29.2)	148 (90.8)	15 (9.2)	0.647 (0.369)	132 (81)	31 (19)	0.065 (3.623)
Specialist	397 (70.8)	342 (89.1)	42 (10.9)		342 (87.2)	50 (12.8)	

*Primary care experience*							
Yes	452 (81.6)	398 (90.2)	43 (9.8)	0.150 (1.476)	386 (86)	63 (14)	0.184 (1.109)
No	102 (18.4)	87 (86.1)	14 (13.9)		81 (81.8)	18 (18.2)	

*Having knowledge about Turkish healthcare system*							
Yes	138 (24.6)	125 (91.9)	11 (8.1)	0.416 (0.880)	121 (87.7)	17 (12.3)	0.407 (0.862)
No	424 (74.4)	368 (89.1)	45 (10.9)		353 (84.4)	65 (15.6)	

*Can adapt to work in refugee health centers?*							
Yes	415 (74.1)	371 (90.9)	37 (9.1)	0.094 (2.221)	355 (86.2)	57 (13.8)	0.223 (0.795)
No/doubtful	145 (25.9)	122 (86.5)	19 (13.5)		118 (83.1)	24 (16.9)	

*Planning to go back to his/her own country?*							
Yes or perhaps	351 (62.5)	301 (87.8)	42 (12.2)	0.082 (3.472	288 (83)	59 (17)	**0.047** (4.396)
No	211 (37.5)	192 (92.8)	15 (7.2)		187 (89.5)	22 (10.5)	

*X* ^2^: chi-square, BDI: Beck Depression Inventory, and BAI: Beck Anxiety Inventory. The values significant at p < 0.05 level are shown in bold.

**Table 6:** The correlation analysis between BDI, BAI, and WHOQOL-BREF domains scores.

		BDI score	BAI score	DOM1 score	DOM2 score	DOM3 score	DOM4 score
BDI score	Rho	1					
	*p* ^*∗*^	—					

BAI score	Rho	0.612	1				
	*p* ^*∗*^	<0.001	—				

DOM1 score	Rho	−0.505	−0.471	1			
	*p* ^*∗*^	<0.001	<0.001	—			

DOM2 score	Rho	−0.555	−0.529	0.692	1		
	*p* ^*∗*^	<0.001	<0.001	<0.001	—		

DOM3 score	Rho	−0.423	−0.393	0.482	0.590	1	
	*p* ^*∗*^	<0.001	<0.001	<0.001	<0.001	—	

DOM4 score	Rho	−0.426	−0.390	0.568	0.590	0.509	1
	*p* ^*∗*^	<0.001	<0.001	<0.001	<0.001	<0.001	—

^*∗*^Spearman correlation. DOM1: domain 1 (physical health), DOM2: domain 2 (psychological), DOM3: domain 3 (social relationships), DOM4: domain 4 (environment), BDI: Beck Depression Inventory, and BAI: Beck Anxiety Inventory.

## Data Availability

The datasets generated and analyzed during the current study are not publicly available due to legal restrictions and are only for use by the research team. If desired, the data can be viewed and reviewed together with the corresponding author.
